# Study on the Low-Temperature Rheology of Polar Drilling Fluid and Its Regulation Method

**DOI:** 10.3390/gels9020168

**Published:** 2023-02-20

**Authors:** Ning Huang, Kaihe Lv, Jinsheng Sun, Jingping Liu, Jintang Wang, Zonglun Wang

**Affiliations:** 1 Department of Petroleum Engineering, China University of Petroleum (East China), Qingdao 266580, China; 2 Key Laboratory of Unconventional Oil & Gas, Development Ministry of Education, Qingdao 266580, China

**Keywords:** polar drilling fluids, low temperature rheology, regulation method, weak gel

## Abstract

Drilling fluid is the blood of drilling engineering. In the polar drilling process, the ultra-low temperature environment puts high demands on the rheological performance of drilling fluids. In this paper, the effects of temperature, ice debris concentration and weighting agent on the rheological properties of drilling fluids were studied. It was found that the lower the temperature and the higher the ice debris concentration, the higher the drilling fluid viscosity, but when the ice debris concentration was below 2%, the drilling fluid rheology hardly changed. Secondly, the low temperature rheological properties of drilling fluid were adjusted by three different methods: base fluid ratio, organoclay, and polymers (dimer acid, polymethacrylate, ethylene propylene copolymer, and vinyl resin). The results showed that the base fluid rheological performance was optimal when the base fluid ratio was 7:3. Compared with polymers, organoclay has the most significant improvement on the low temperature rheological performance of drilling fluid. The main reason is that organoclay can transform the drilling fluid from Newtonian to non-Newtonian fluid, which exhibits excellent shear dilution of drilling fluid. The organoclay is also more uniformly dispersed in the oil, forming a denser weak gel mesh structure, so it is more effective in improving the cuttings carrying and suspension properties of drilling fluids. However, the drilling fluid containing polymer additives is still a Newtonian fluid, which cannot form a strong mesh structure at ultra-low temperatures, and thus cannot effectively improve the low-temperature rheological performance of drilling fluid. In addition, when the amount of organoclay is 2%, the improvement rate of the yield point reaches 250% at −55 °C, which can effectively improve the cuttings carrying and suspension performance of drilling fluid at ultra-low temperature.

## 1. Introduction

In recent years, with the increasing national demand for energy, oil and gas exploration has gradually expanded to deep land, deep sea, and complex strata, such as polar regions. Antarctica is rich in resources, with an area of 14.5 million km^2^. However, 99% of the area is covered by ice, and the average thickness of the ice cap is 1829 m. Antarctica is rich in mineral resources, with 220 types of proven minerals, including coal, oil, natural gas, diamond, and uranium of strategic importance. It is estimated that the current oil reserves in the Antarctic region are about (7.95–15.90) × 10^9^ m^3^, with natural gas reserves of about (3–5) × 10^12^ m^3^, and the potential resources of hydrates at the land edge are about (4.7–7.8) × 10^13^ m^3^. However, due to the low Antarctic surface temperature (−60 to −50 °C) [1,2], the complex geological environment under the ice (divided into snow, ice, and rock layers from top to bottom) and the large variation of formation temperature (−55 to −2 °C), this poses a huge challenge to the performance of drilling fluid. Drilling fluid is the blood of drilling engineering. High-performance low-temperature drilling fluid is an important guarantee for safe and efficient drilling in deep ice formations and ice-rock interburden. Among them, the regulation of drilling fluid rheological performance will be one of the key factors to restrict downhole safety and affect the drilling speed in the drilling process.

At present, research scholars have conducted a lot of studies on the rheological properties of drilling fluids under high temperature, high pressure [3,4,5,6], and deep water [7] conditions, and certain research results have been achieved. However, there are relatively few studies on polar cryogenic drilling fluids at home and abroad. At present, only a few cryogenic drilling materials have been selected for deep ice core drilling, such as petroleum-based drilling fluids [8,9] and alcohol-based drilling fluids [10,11]. The European Project for Ice Cores in Antarctica (EPICA) team used ethanol aqueous solution as drilling fluid for ice core drilling at Dome C, Antarctica. However, it was found to corrode the ice cores and affect the quality of the ice cores [12]. The United States Ice Drilling Program (IDP) used isoparaffinic solvent IsoparK as drilling fluid in Western Antarctica from 2016 to 2017 [13]. In addition, with the increase in research, new materials, such as esters [14,15] and silicone oil [16] have also been successfully used as drilling fluids for polar deep ice coring operations. China used butyl acetate to drill 800.8 m and successfully drilled 337 ice cores from 2009–2017 in Dome A, at the East Antarctic Plateau [17,18]. Some researchers also used the highly viscous ESTISOL-240/COASOL drilling fluid [19] in Northwest Greenland to drill 2537.36 m. However, the ultra-low temperature led to a significant increase in the viscosity of the drilling fluid and the strength of the gel, thereby making the drill bit stuck, leading to the end of the study. However, all of the above studies only conducted ice core drilling tests using different materials, and there is a lack of published research on the rheology of polar drilling fluids. In addition, drilling fluids for high temperatures, pressure, and deep water are basically non-Newtonian fluids with good shear dilution properties. In contrast, the polar drilling fluids reported so far are basically Newtonian fluids with a small yield point and poor rheological properties. Therefore, research scholars need to make efforts to improve the rheological properties of polar drilling fluids.

The rheological performance of drilling fluids at ultra-low temperatures is one of the key issues in the polar drilling process. However, when there are deficiencies in the rheological performance regulation of the drilling fluid, the main problems are low yield point and poor suspension ability. These problems tend to make the drilling fluid carry the cuttings unfavorably, causing problems, such as cuttings accumulation at the bottom of the well, abnormal agitation pressure, and equivalent circulation density, leading to downhole accidents and ultimately affecting drilling costs. The excellent rheological performance is directly related to the efficiency of drilling fluid in carrying cuttings, and indirectly affects the drilling speed. However, there are no reports on the rheological performance of polar drilling fluids at home and abroad. Therefore, in view of the above problems, this paper has studied the low temperature rheology law of polar drilling fluid and the rheology adjustment methods. In addition, the influence mechanism of various adjustment methods on the rheology of drilling fluids was also analyzed. It has certain reference significance for further polar drilling.

## 2. Results and Discussion

### 2.1. Study on the Influence Law of Low Temperature Rheology of Drilling Fluids

Following the investigation into the research status of polar ultra-low temperature drilling fluids, the influence law of temperature, concentration of ice debris, and weighting agent addition on the rheology of polar drilling fluids (aviation kerosene) were explored in depth.

#### 2.1.1. Effect of Temperature on the Rheology of Aviation Kerosene-Based Fluid

Temperature is an important factor affecting the rheology of drilling fluids. The influence of temperature on the rheology of aviation kerosene drilling fluid is revealed by exploring the change of its viscosity at different temperatures.

**Figure 1:**
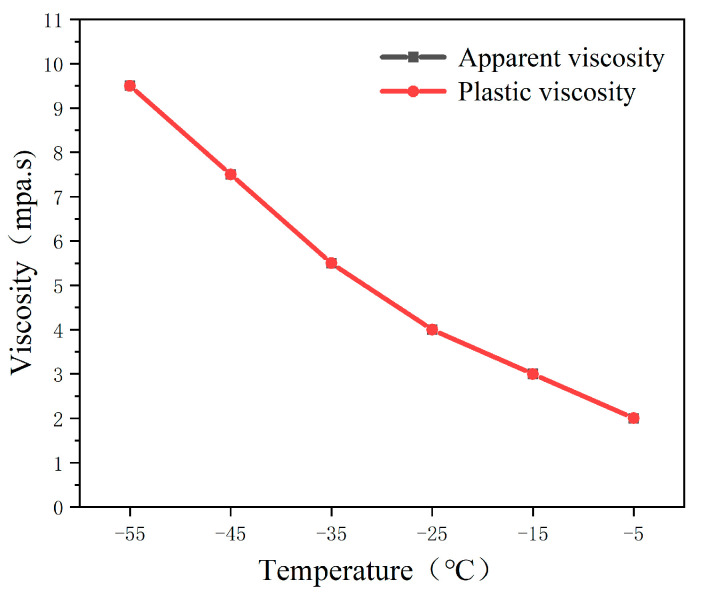
Influence of temperature on the viscosity of drilling fluid.

Experimental studies showed that the viscosity of drilling fluid gradually increased as the temperature gradually decreased in the range of −55 °C to −5 °C, with an obvious low-temperature thickening phenomenon (as shown in Figure 1). This may be because the intermolecular distance of aviation kerosene decreases as the temperature decreases, which makes the intermolecular force increase, and then the viscosity gradually increases. in addition, it may also be caused by the sudden change in the flexibility of molecules due to the change in temperature.

#### 2.1.2. Effect of Ice Debris Concentration on the Rheology of Aviation Kerosene-Based Fluid

When drilling into the ice, the drilling fluid will face the invasion of ice debris. However, the invasion of ice debris will have a certain effect on the rheology of the drilling fluid. Therefore, it is very important to explore the influence of ice debris concentration on the rheology of the drilling fluids for safe and efficient drilling. This section explores the effect of the concentration of ice debris on the rheological properties of drilling fluid at −55 °C with a specific particle size of ice debris (0.1–0.3 mm). The addition of ice debris is 0%, 2%, 4%, 6%, and 8%, respectively.

**Figure 2:**
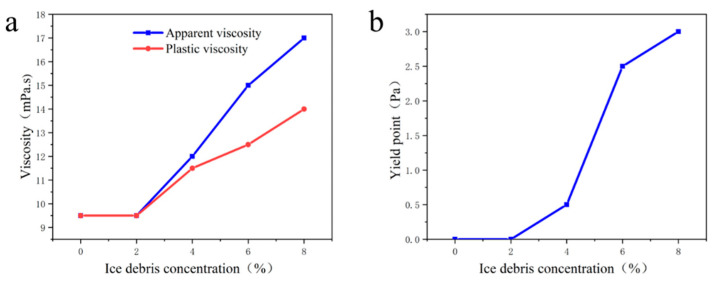
Influence of ice debris concentration on the rheological properties of drilling fluid; (**a**) viscosity; (**b**) yield point.

The experimental study found that the viscosity and yield point of drilling fluid gradually increased with the increasing concentration of ice cuttings (Figure 2). When the concentration of ice debris was below 2%, the rheological properties of the drilling fluid hardly changed. It may be that when the concentration of ice debris is in the low concentration range (0~2%), the collision probability of ice debris particles under shear action is very small, and the friction between particles is very weak, so the viscosity and yield point of drilling fluid do not change. However, when the concentration of ice debris is higher than 2%, the collision probability of ice debris particles under shear stress gradually increases with the increase of the concentration of ice debris, and the friction between particles is enhanced, which leads to the gradual increase of the viscosity of the drilling fluid and thus the increase of the yield point of the drilling fluid. In addition, it has been suggested that drilling fluid viscosity below 25 mpa·s has the least effect on drilling when drilling in ice formations, and the lower the viscosity, the better [20]. The above study shows that this ice debris concentration is acceptable for drilling.

#### 2.1.3. Effect of Weighting Agent Addition on the Rheology of Aviation Kerosene-Based Fluid

The experimental study found that the density of aviation kerosene-based fluid is 0.78 g/cm^3^, while the density required for polar drilling fluid should be 0.92–0.95 g/m^3^. Therefore, it is necessary to increase the drilling fluid density by adding a weighting agent. In addition, the smaller the viscosity of the required drilling fluid, the better the drilling speed.

**Figure 3:**
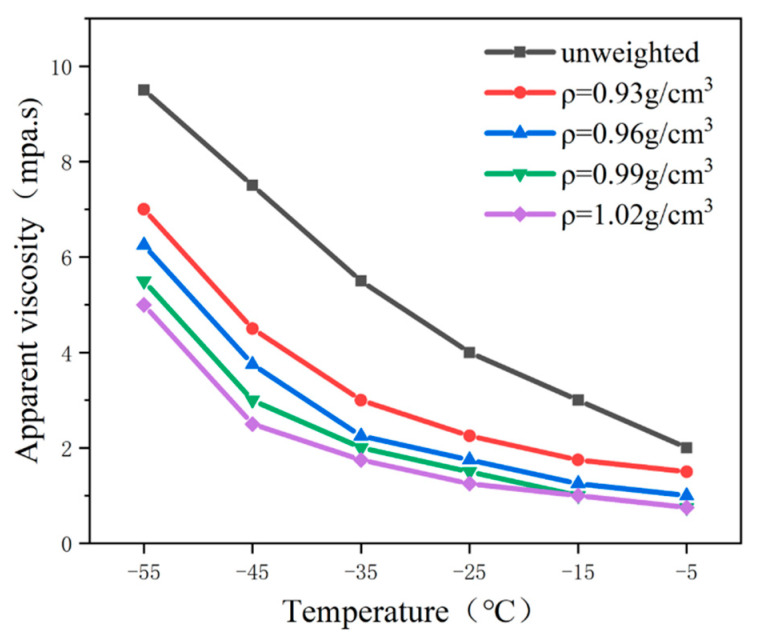
Influence of the weighting agent addition on the apparent viscosity of the drilling fluid.

To investigate the effect of the weighting agent addition on the rheological properties of aviation kerosene drilling fluid, HCFC-141b weighting agent was used to adjust to the different densities. The viscosity-temperature characteristics of the aviation kerosene drilling fluid were studied at different weighting densities (0.93, 0.96, 0.99, 1.02 g/cm^3^), and the results are shown in Figure 3.

From Figure 3, it can be seen that the viscosity of the drilling fluid gradually decreases with the increase of density at different temperatures. At the same density, the drilling fluid viscosity also gradually increases with the decrease in temperature. In addition, when the drilling fluid density gradually increases, the drilling fluid viscosity will also gradually decrease, but the drilling fluid viscosity will finally tend to stabilize and remain basically the same. This is due to the fact that the viscosity of the weighting agent at an ultra-low temperature is much smaller than that of aviation kerosene. Therefore, the viscosity of aviation kerosene-based fluid is significantly reduced by the addition of the weighting agent.

### 2.2. Study on the Rheological Regulation Method of the Drilling Fluid at Low Temperature

Currently, aviation kerosene is often used as drilling fluid during drilling in the Antarctic region. However, when aviation kerosene is used as the base fluid, it exhibits the problems of poor cuttings carrying and suspension properties. In this paper, we try to adjust the rheological performance of drilling fluid at ultra-low temperatures by adding white oil to adjust the ratio of the base fluid, adding organoclay and polymers with different molecular structures. The main index is the yield point of drilling fluid to improve the cuttings carrying and suspension performance at ultra-low temperature.

#### 2.2.1. Base Fluid Ratio Adjustment

**Figure 4:**
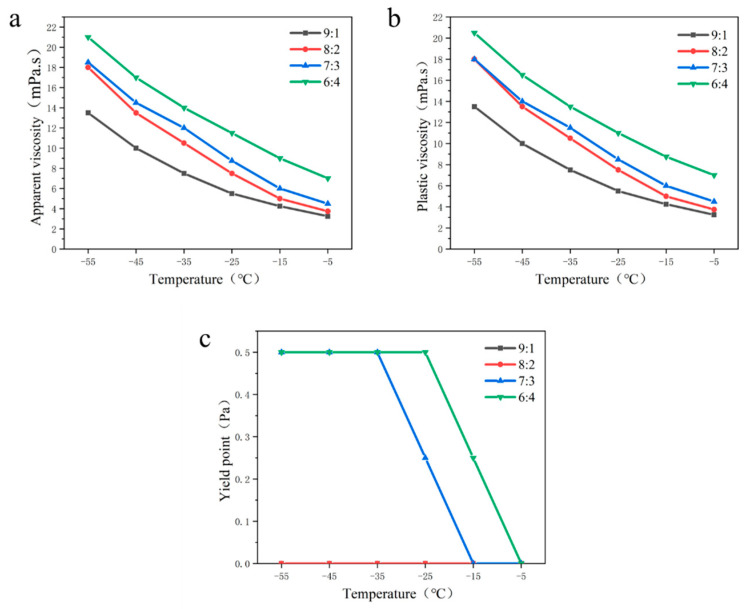
Influence of the base fluid ratio on the rheological properties of the drilling fluid; (**a**) apparent viscosity; (**b**) plastic viscosity; (**c**) yield point.

The molecular structure of no. 5 white oil is similar to that of aviation kerosene, and its effect on the low-temperature rheological properties of drilling fluid is studied by adding different ratios of white oil. The rheological properties of drilling fluid may be changed with the change of base fluid ratio. Aviation kerosene and white oil were mixed in different ratios (9:1, 8:2, 7:3, 6:4) to explore the effect of the base fluid ratios on the drilling fluid rheology at different temperatures. The experimental results are shown in Figure 4.

It can be seen from Figure 4a,b that the apparent viscosity and plastic viscosity of the drilling fluid increase as the ratio of the base fluid decreases. When the base fluid ratio was 6:4, the apparent viscosity and plastic viscosity of the drilling fluid showed the maximum at any temperature. This indicates that as the proportion of white oil increases, the viscosity of drilling fluid at low temperatures increases. The results show that the viscosity-temperature characteristics of the drilling fluid changed greatly with the addition of white oil. However, when the ratio of the base fluid was 7:3, the viscosity of drilling fluid gradually increased with the decrease of temperature, but the viscosity was lower than that of the drilling fluid when the ratio of the base fluid was 6:4 at any temperature. In addition, with the decrease of temperature, the viscosity of drilling fluid under different base fluid ratios increased gradually.

According to Figure 4c, when the base fluid ratio was 9:1 and 8:2, the yield point of the drilling fluid did not change with the temperature. It showed that this proportion of white oil had no effect on the yield point of the drilling fluid. When the ratio of the base fluid increased to 7:3 and 6:4, the yield point of drilling fluid was 0.5 Pa at −55 °C, and the yield point was the best. It showed that the internal structural force of drilling fluid was the strongest at this time, and the influence of temperature change was the weakest.

In conclusion, the yield point of drilling fluid with a base fluid ratio of 7:3 and 6:4 is the same at an ultra-low temperature (≤−35 °C), but the viscosity of the base fluid at 7:3 is smaller at an ultra-low temperature, which is more suitable for Antarctic drilling requirements. Therefore, when the base fluid ratio is 7:3, the rheological property of the drilling fluid is the best.

#### 2.2.2. Study on the Rheological Regulation of Organoclay

Organoclay is commonly used as a rheology modifier for oil-based drilling fluids [21,22] and has significant rheology modifying effects. However, there are no reports on the application of organoclay in polar drilling fluid research. For this reason, the effect of organoclay (Baker’s commercial clay) on the rheology of polar drilling fluids was investigated by selecting organoclay as an additive. The experimental study found that the addition of organoclay alone to the base fluid of polar drilling fluid resulted in significant settling and poor gel formation performance. For this reason, a certain amount of amide emulsifier (Hanke emulsifier) was added to the experiment to improve the low-temperature gelation rate of organoclay in the base fluid.

To study the effect of organoclay on the rheology of ultra-low temperature drilling fluid, organoclay (Baker’s commercial clay) and amide emulsifier (Hanke emulsifier) were added on the basis of 7:3 base fluid ratio to formulate ultra-low temperature drilling fluid, and the effect of different concentrations of organoclay on the rheological performance of the drilling fluid was investigated separately.

**Figure 5:**
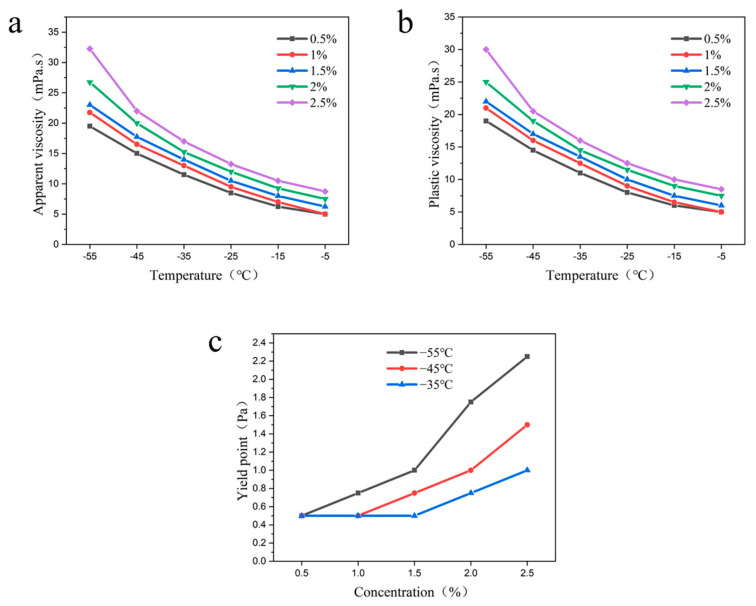
Effects of the different concentrations of organoclay on the rheological properties of the drilling fluid; (**a**) apparent viscosity; (**b**) plastic viscosity; (**c**) yield point.

The experimental study found that the apparent viscosity, plastic viscosity, and yield point of the drilling fluid showed a gradual increase with the increase of organoclay concentration at a specific temperature (Figure 5). At a certain addition amount, the drilling fluid viscosity and yield point also showed a gradual increase with the decreasing temperature. In addition, it was also found that the yield point gradually increased with the increase of organoclay concentration under any specific temperature condition. This indicates that organoclay can effectively improve the low-temperature rheological properties of drilling fluid. The analysis may be due to the joint action of organoclay particles and emulsifier molecules through hydrogen bonding, emulsifier molecules through intermolecular forces and ultra-low temperature to form a weak gel mesh structure inside the drilling fluid, which enhances the internal structural force of the drilling fluid and thus effectively improves the rheological properties of the drilling fluid. In addition, the experimental study showed that the plastic viscosity of the drilling fluid at −55 °C was 25 mpa·s and the yield point was 1.75 Pa when the amount of organoclay was 2%, and the improvement rate of the yield point was 250%.

#### 2.2.3. Influence of Polymer on Rheological Properties of Drilling Fluid

According to Section 2.2.1, the rheological property of drilling fluid is optimal when the ratio of the base fluid is 7:3. Therefore, on the basis of the above studies, four polymers with different molecular structures, namely dimeric acid [23,24,25,26], polymethacrylate [27], vinyl resin [28], and ethylene propylene copolymer [29], were added, respectively, to study their effects on the rheological properties of drilling fluids.

1. Dimeric Acid

Different concentrations of dimeric acid (0.5%, 1%, 1.5%, 2%, 2.5%) were added to the base fluid of drilling fluid to explore the effects of different concentrations on the apparent viscosity, plastic viscosity, and yield point of the drilling fluid at −55 °C~−5 °C.

**Figure 6:**
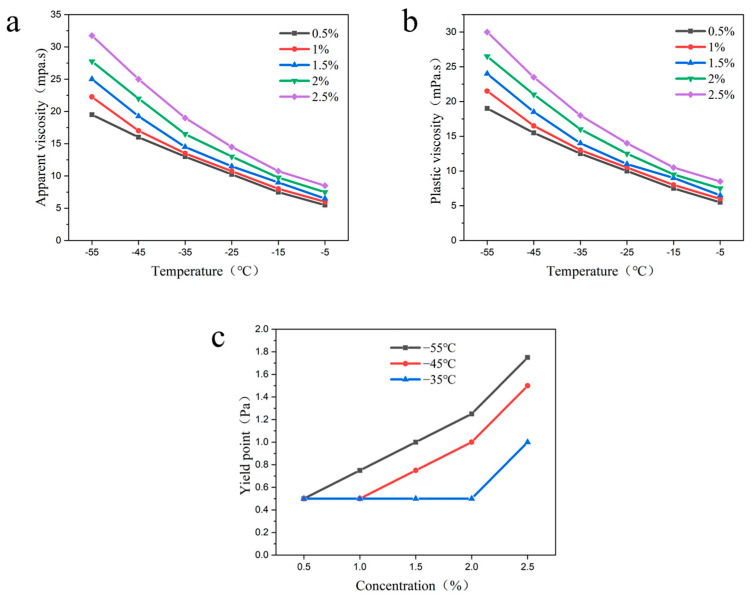
Effects of dimeric acid at different concentrations on the rheological properties of drilling fluid; (**a**) apparent viscosity; (**b**) plastic viscosity; (**c**) yield point.

It was found that the apparent viscosity, plastic viscosity, and yield point of the drilling fluid showed a gradual increase with increasing dimeric acid concentration at the same temperature (Figure 6). This may be due to the increase of dimeric acid concentration, which makes the intermolecular interaction force and hydrogen bonding gradually increase to form a strong grid structure. This makes the drilling fluid viscosity and yield point exhibit a gradual increase. In addition, at a certain concentration, the viscosity and yield point of the drilling fluid increase with the decrease in temperature. This is because the decrease in temperature reduces the distance between molecules, resulting in the increase of intermolecular force, and then the viscosity and yield point gradually increase, which enhances the structural force of the drilling fluid. In addition, the experimental study showed that the plastic viscosity of the drilling fluid at −55 °C was 26.5 mpa·s and the yield point was 1.25 Pa when dimeric acid was added at 2%, and the improvement rate of the yield point was 150%.

2. Polymethacrylate

In this section, the effects of different concentrations of polymethacrylate (0.5%, 1%, 1.5%, 2% and 2.5%) on the apparent viscosity, plastic viscosity, and yield point of the drilling fluid were investigated at −55 °C to −5 °C.

**Figure 7:**
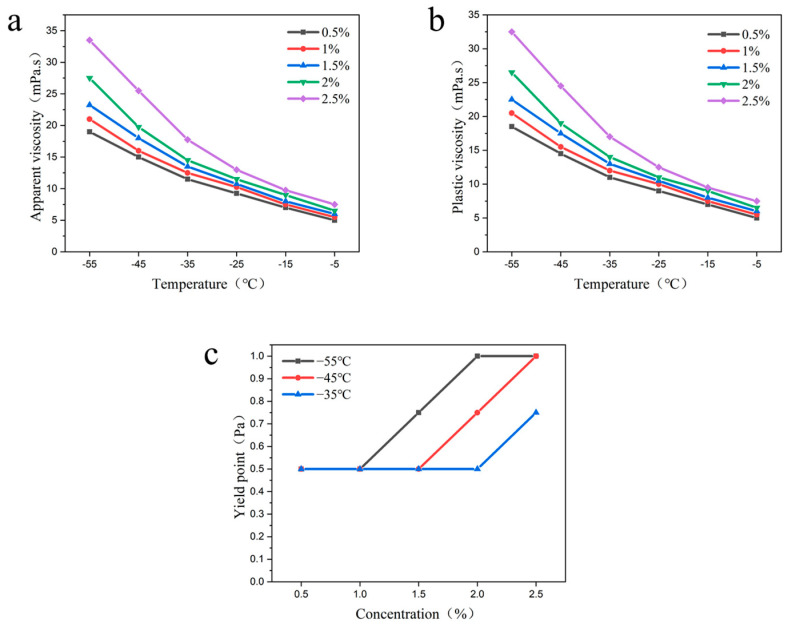
Effects of the different concentrations of polymethacrylate on the rheological properties of the drilling fluid; (**a**) apparent viscosity; (**b**) plastic viscosity; (**c**) yield point.

It was found that the apparent viscosity, plastic viscosity, and yield point of the drilling fluid showed a gradual increase with increasing polymethacrylate concentration at the same temperature (Figure 7). This may be due to the fact that as the concentration of polymethacrylate increases, the intermolecular force increases due to the physical entanglement between the molecular chains, which results in a stronger mesh structure. In addition, when the concentration of polymethacrylate was below 2%, the yield point of the drilling fluid did not change significantly with the decrease in temperature. In contrast, when the concentration was higher than 2%, the drilling fluid yield point gradually increased with the decreasing temperature. This may be due to the fact that at low concentrations, the enhancement of intermolecular van der Waals forces is not obvious due to the decrease in temperature, which in turn shows that the yield point does not change basically. In addition, the experiments showed that the plastic viscosity of the drilling fluid was 26.5 mpa·s and the yield point was 1 Pa at −55 °C when the dosage of polymethacrylate was 2%, and the improvement rate of yield point was 100%.

3. Ethylene Propylene Copolymer

Different concentrations of ethylene propylene copolymer (0.5%, 1%, 1.5%, 2%, 2.5%) were added to the drilling fluid to investigate the effects of different concentrations of ethylene propylene copolymer on the apparent viscosity, plastic viscosity, and yield point of the drilling fluid under the conditions of −55 °C~−5 °C.

**Figure 8:**
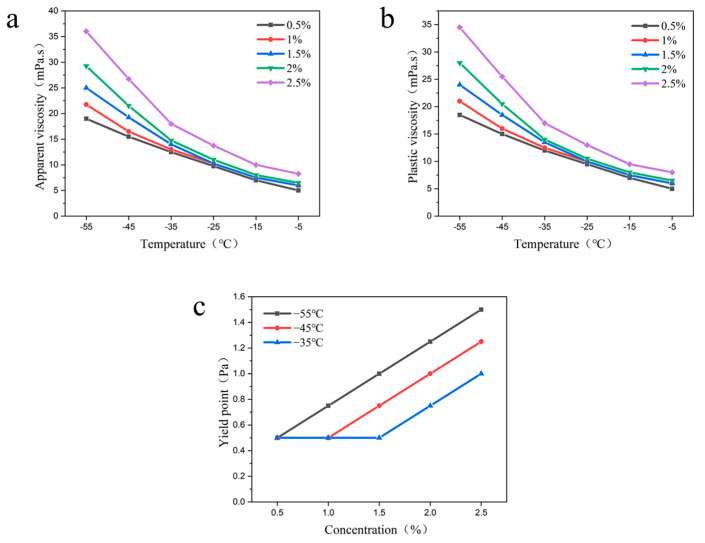
Effects of the different concentrations of ethylene propylene copolymer on the rheological properties of drilling fluid; (**a**) apparent viscosity; (**b**) plastic viscosity; (**c**) yield point.

The experimental study found that the apparent viscosity, plastic viscosity, and yield point of drilling fluid showed a gradual increase with the increase of ethylene propylene copolymer concentration at a specific temperature (Figure 8). The drilling fluid viscosity and yield point also showed a gradual increase with the decrease in temperature at a certain addition amount. In addition, it was found that the increase in drilling fluid viscosity became larger when the addition amount of ethylene propylene copolymer was increased to 2.5%, while the increase in yield point remained the same. This may be due to the high concentration of polymer molecular chains to agglomerate and entangle, making the viscosity increase larger. The reason for the constant increase in the yield point may be that after the formation of an effective grid structure, the excess ethylene propylene copolymer molecules just accumulate in a disorderly manner, and thus the increase in yield point exhibits a stable state. In addition, experimental research showed that when the amount of ethylene propylene copolymer addition was 2%, the plastic viscosity of the drilling fluid at −55 °C was 28 mpa·s, with the yield point of 1.25 Pa, and the improvement rate of yield point was 150%.

4. Vinyl Resin

**Figure 9:**
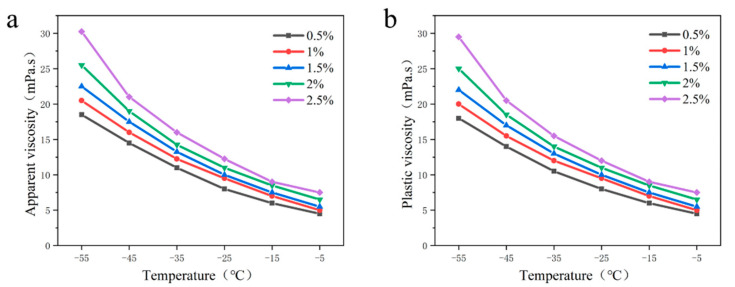
Effects of the different concentrations of vinyl resin on the rheological properties of drilling fluid; (**a**) apparent viscosity; (**b**) plastic viscosity.

The effects of different concentrations of vinyl resin (0.5%, 1%, 1.5%, 2%, 2.5%) on the apparent viscosity and plastic viscosity of drilling fluid at specific temperatures and the relationship between the viscosity of drilling fluid and temperature at specific additions of vinyl resin were investigated, respectively. The experimental study found that the apparent viscosity and plastic viscosity of the drilling fluid showed a gradual increase with the increase of vinyl resin concentration at the same temperature (Figure 9). The yield point basically did not change. It showed that the intermolecular force of vinyl resin was very weak at low temperatures, which did not form an effective mesh structure and thus did not improve the yield point. In addition, the viscosity of the drilling fluid increased with the decrease of temperature at a certain additive amount, which also confirmed the conclusion that the decrease in temperature would increase the intermolecular van der Waals force.

By studying the influence of polymers on the rheology of drilling fluid, it was found that the drilling fluid containing dimeric acid had the best low-temperature rheological performance. The plastic viscosity of the drilling fluid with 2% dimeric acid was 26.5 mpa·s at −55 °C, and the yield point was 1.25 Pa, with a 150% improvement in the yield point. However, The low-temperature rheological properties of the drilling fluid improved by the vinyl resin were the weakest. The plastic viscosity of the drilling fluid with 2% vinyl resin was 25 mpa·s at −55 °C, and the yield point was 0.5 Pa, with 0% improvement in the yield point.

The study on the rheological regulation method of drilling fluid at low temperatures showed that the rheological property of drilling fluid at ultra-low temperatures was the best when the ratio of the base fluid was 7:3. When organoclay and four different polymers (dimeric acid, polymethacrylate, ethyl-propylene copolymer, and vinyl resin) were added at 2%, the yield point of drilling fluid containing organoclay was the highest, which could effectively improve the cuttings carrying and suspension performance of the drilling fluid at ultra-low temperatures.

On this basis, the viscosity variation of the drilling fluids with different additives at low shear rates and the variation of shear stress with shear rate were further investigated by means of a Haake rheometer.

**Figure 10:**
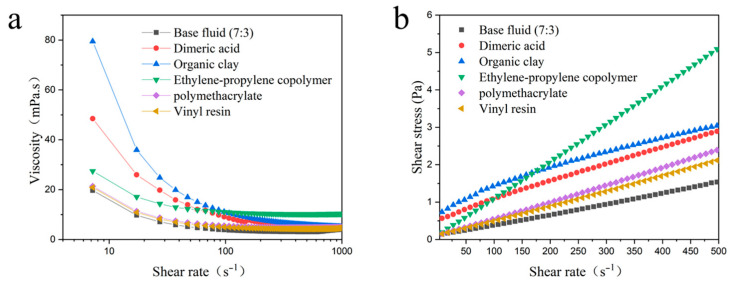
Haake rheological curve of drilling fluid with 4% different additives; (**a**) viscosity with shear rate; (**b**) graph of shear stress variation with shear rate.

As shown in Figure 10a, the variation of viscosity with a shear rate for drilling fluids containing different additives was investigated after freezing at −55 °C for 16 h and measured at 2 °C. It was shown that at lower shear rates, the viscosity of the drilling fluids containing organoclay decreased significantly with increasing shear rate, indicating excellent non-Newtonian properties of shear dilution. However, for drilling fluids incorporating other additives, as well as the 7:3 base fluid, the viscosity changes slowly with the increasing shear rate. It indicates that drilling fluids containing organoclay are more favorable for cuttings carrying at ultra-low temperatures.

From Figure 10b, it can be seen that the shear stress of the drilling fluid containing organoclay shows a nonlinear relationship with the variation of shear rate, indicating that the curve is consistent with the non-Newtonian model. In contrast, the variation relationship curve for drilling fluids containing other additives is linear, which is consistent with the Newtonian model. When the drilling fluid exhibits a non-Newtonian model, it will have a higher yield point. This indicates that a mesh structure of a certain strength is formed inside the drilling fluid, which can effectively improve the cuttings carrying and suspension performance of the drilling fluid and ensure the cleanliness of the borehole. The above study shows that the addition of organoclay transforms the drilling fluid from a Newtonian fluid to a non-Newtonian fluid, possessing shear dilution and improving the rheological properties of the drilling fluid. This is consistent with the results of the study on the relationship between viscosity and shear rate.

### 2.3. Analysis of the Rheological Regulation Mechanism

The influence mechanism of the different regulation methods on the rheology of drilling fluid at low temperatures was studied. The mechanism of rheology regulation was analyzed by observing the microscopic dispersion state and morphology.

**Figure 11:**
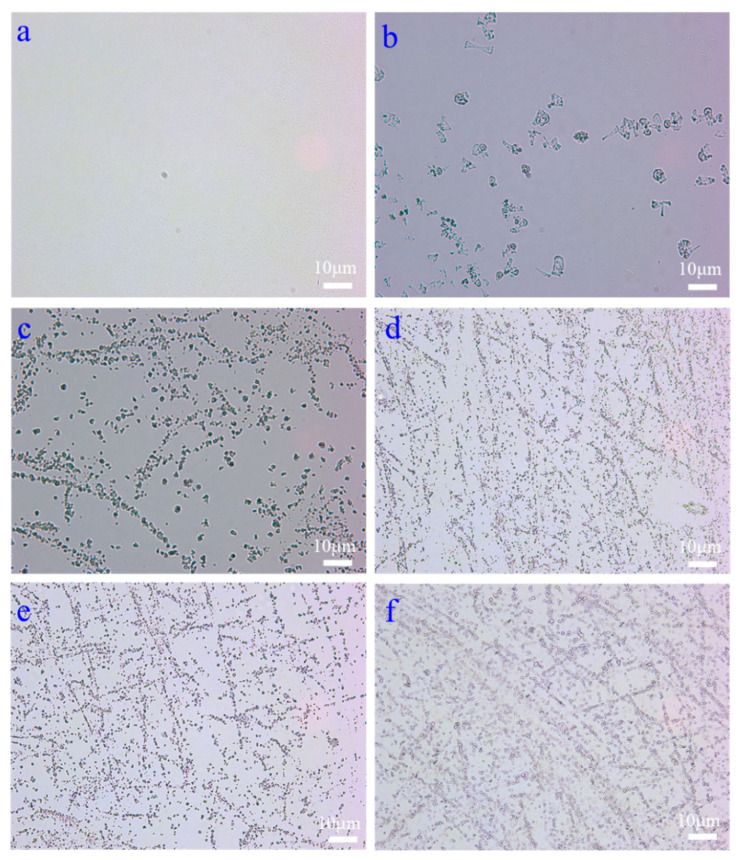
Optical microscope images of the drilling fluid containing 4% different additives after freezing at −55 °C for 16 h; (**a**) 7:3 base fluid; (**b**) vinyl resin; (**c**) polymethacrylate; (**d**) ethylene propylene copolymer; (**e**) dimeric acid; (**f**) Baker’s organoclay.

As can be seen from Figure 11, the drilling fluid containing vinyl resin could not be uniformly dispersed in the oil after freezing at −55 °C, and the vinyl resin molecules appeared to aggregate in lumps, indicating its poor lipophilicity at low temperatures. In addition, the vinyl resin also did not form a mesh structure, which in turn could not improve the rheological properties of the drilling fluid. Compared with vinyl resins, the dispersion of polymethacrylate, ethylene propylene copolymer, and dimeric acid in oil improved overall. Locally, ethylene propylene copolymer and dimeric acid formed a mesh structure in the drilling fluid, while polymethacrylate basically did not form a mesh structure in the drilling fluid. It can be concluded from the figure that organoclay is more uniformly dispersed in the oil and forms a more dense mesh structure. The analysis may be due to the joint action of organoclay particles and emulsifier molecules through hydrogen bonding, emulsifier molecules through intermolecular forces, and ultra-low temperature to form a weak gel mesh structure inside the drilling fluid, which enhances the internal structural force of the drilling fluid and thus effectively improves the rheological properties of the drilling fluid.

### 2.4. Future Works with Applicability

At present, the research on the rheological performance of polar drilling fluids is still basically in its infancy. In this paper, the rheological laws and adjustment methods of polar drilling fluids were studied to understand the difficult problem of low yield point and poor suspension ability of polar drilling fluids. It was shown that organoclay was the best rheological regulator for polar drilling fluids, which could effectively improve the low temperature rheological performance of drilling fluids. The four different polymers were not satisfactory for improving the rheological properties of the drilling fluids. Therefore, in future polar drilling processes, organoclay can be added to the drilling fluid to improve the low-temperature rheological properties of the drilling fluid and thus improve the drilling efficiency. This study also has a certain contribution to the development of polar drilling.

## 3. Conclusions

The rheological performance of drilling fluid is one of the key factors governing downhole safety and affecting drilling speed during drilling. In this paper, the effects of temperature, ice debris concentration, and weighting agent on the rheological properties of drilling fluid were investigated. It was found that the lower the temperature and the higher the concentration of ice debris, the higher the viscosity of the drilling fluid. However, when the ice debris concentration was below 2%, the rheological properties of the drilling fluid did not change. The drilling fluid viscosity decreased as the weighting agent was added, and the decrease in drilling fluid viscosity reduced as the weighting agent was added. Secondly, the study of the low-temperature rheological adjustment method of drilling fluid showed that the best rheological performance of drilling fluid base fluid at ultra-low temperature was achieved when the ratio of the base fluid was 7:3. Among the organoclay and four different polymers (dimeric acid, polymethacrylate, ethylene propylene copolymer, and vinyl resin) rheology regulators, organoclay was the best ultra-low temperature rheology regulator. The main reason for this is that organoclay is able to transform drilling fluid from Newtonian to non-Newtonian fluid and exhibit excellent shear dilution of the drilling fluid. Moreover, organoclay is more uniformly dispersed in the oil and forms a denser weak gel mesh structure. Therefore, it is more effective in improving the cuttings carrying and suspension performance of drilling fluid. However, the drilling fluid containing polymer additives is still a Newtonian fluid, which cannot form a strong mesh structure at ultra-low temperatures. Therefore, they cannot effectively improve the low-temperature rheological performance of drilling fluid. In addition, when the amount of organoclay is 2%, the improvement rate of the yield point reaches 250% at −55 °C, which can effectively improve the cuttings carrying and suspension performance of the drilling fluid at ultra-low temperatures. The results of this study have some important implications for polar oil and gas exploration and development.

## 4. Materials and Methods

### 4.1. Materials

No. 4 aviation kerosene (purity > 98%) was purchased from Jinan Xiangtai Chemical Co., Ltd. in Jinan, China. No. 5 QY white oil (purity > 99%) was purchased from Guangdong Maoming Petrochemical Co., Ltd. in Guangdong, China. Dimeric acid (purity > 98%) and vinyl resin (purity > 98%) were purchased from Shanghai Zhipu Chemical Co., Ltd. in Shanghai, China. Polymethacrylate (purity > 85%) and ethylene propylene copolymer (purity > 85%) were purchased from Jinzhou Runzhifeng Chemical Co., Ltd. In Jinzhou, China. Baker’s commercial organoclay was purchased from Baker Petroleum Technology Development Services Co., Ltd. in Yanan, China. Monofluorodichloroethane (HCFC-141b) was purchased from Shandong Yuke Chemical Co., Ltd. Emulsifier was purchased from Hanke Petroleum Technology Co., Ltd. in Shandong, China. The emulsifier (purity > 98%) was from Chengdu Hanke Petroleum Technology Co., Ltd. in Chengdu, China.

### 4.2. Experimental Methods

#### 4.2.1. Influence Law of Low Temperature Rheology of the Drilling Fluid

1. Temperature

The rheological properties of aviation kerosene-based fluids at different temperatures are measured by using a new integrated ultra-low temperature circulating ZNN-D6 electronic viscometer (Tongchun, Qingdao, China). The device can be used to derive the apparent viscosity, plastic viscosity, and yield point of the drilling fluid at different temperatures, so as to judge the advantages and disadvantages of the ultra-low temperature rheological performance of the drilling fluid.

A certain amount of aviation kerosene was poured into the measuring cylinder, and the temperature was controlled by the thermostat system of the above instrument to measure the size of aviation kerosene base fluid Φ600 and Φ300 at different temperatures (−5 °C, −15 °C, −25 °C, −35 °C, −45 °C, −55 °C), respectively. The apparent viscosity, plastic viscosity, and yield point were calculated by Equations.

AV = 0.5 · θ600(1)

PV = θ600 − θ300(2)

YP = 0.5·(θ300 − PV)(3)

where AV is the apparent viscosity (mPa·s), PV is the plastic viscosity (mPa·s), and YP is the yield point (Pa). In addition, Φ600 and Φ300 refer to 600 r/min and 300 r/min in ZNN-D6 electronic viscometer respectively, which are two different shear rates.

2. Ice Debris Concentration

Ice debris of specific particle size (0.1–0.3 mm) were added into aviation kerosene to investigate the effect of ice debris concentration on the rheological properties of aviation kerosene-based fluid. Deionized water was first frozen in a DW-40 ultra-low temperature thermostat (Xinxing, Cangzhou, China), and then ice debris of specific particle size (0.1–0.3 mm) were made in an SJ-M502S ice crusher (Serno, Guangdong, China). The ice debris with different additive amounts were added to aviation kerosene separately, and the sizes of drilling fluids Φ600 and Φ300 containing different ice debris concentrations (0%, 2%, 4%, 6%, 8%) at −55 °C were measured by a new integrated ultra-low temperature circulating ZNN-D6 electronic viscometer. the apparent viscosity, plastic viscosity, and yield point were calculated by equations.

3. Aggravator Dosing

HCFC-141b was selected as the drilling fluid aggravating agent, and different amounts of aggravating agent were added to the aviation kerosene separately. the drilling fluid with different densities (0.93, 0.96, 0.99, 1.02 g/cm^3^) was prepared by stirring with ZMD-550 high-speed stirrer (Tongchun, Qingdao, China) for 15~20 min at room temperature. The variation of apparent viscosity with the temperature (−5 °C, −15 °C, −25 °C, −35 °C, −45 °C, −55 °C) of aviation kerosene-based fluids of different densities was measured by the new integrated ultra-low temperature cycle ZNN-D6 electronic viscometer.

#### 4.2.2. Drilling Fluid Low-Temperature Rheology Adjustment Method

1. Base Fluid Ratio Adjustment

The base fluid was prepared by mixing aviation kerosene and white oil in different ratios (9:1, 8:2, 7:3, 6:4) and stirred for 25~30 min with ZMD-550 high-speed stirrer (Qingdao Tongchun, China), and then the effects of the base fluid ratios on the drilling fluid rheological parameters (AV, PV, YP) were measured at different temperatures.

2. Organoclay Rheology Adjustment

On the basis of the base fluid (7:3), 3% of Hankook emulsifier was added and stirred for 15~20 min with ZMD-550 high-speed stirrer (Tongchun, Qingdao, China), followed by adding different concentrations of organoclay and continuing to stir for 15~20 min to prepare for polar drilling fluid. The changes of rheological parameters (AV, PV, YP) of drilling fluid containing different concentrations of organoclay with temperature were measured by a new integrated ultra-low temperature circulation ZNN-D6 electronic viscometer.

3. Polymer Regulation of Rheological Properties of Drilling Fluids

On the basis of the base fluid (7:3), polymers with different molecular structures (dimeric acid, polymethacrylate, ethylene propylene copolymer, vinyl resin) were added and stirred for 25~30 min with a ZMD-550 high-speed stirrer (Tongchun, Qingdao, China) to prepare the drilling fluids containing different polymers. The changes of the rheological parameters (AV, PV, YP) with the temperature of drilling fluids containing different polymers were measured separately using a new integrated ultra-low temperature cycle ZNN-D6 electronic viscometer.

The base fluid (7:3), drilling fluids containing 4% organoclay, and different polymers (4%) were frozen (−55 °C) for 16 h in a DW-40 ultra-low temperature thermostat (Xinxing, Cangzhou, China), and then the changes in the viscosity of the drilling fluids at low shear rates were measured by a Haake MARS rheometer (Thermo Fisher Scientific, Waltham, MA, USA) at 2 °C, and the relationship between the shear stress and shear rate of the drilling fluid was measured at 2 °C. The Haake MARS rheometer can analyze whether the drilling fluid has shear dilution properties and can determine whether the drilling fluid is a Newtonian or non-Newtonian fluid.

#### 4.2.3. Mechanistic Analysis

1. Optical Microscope Analysis

The base fluid (7:3), drilling fluids containing 4% organoclay, and different polymers (4%) were frozen (−55 °C) for 16 h in a DW-40 ultra-low temperature thermostat (Xinxing, Cangzhou, China), and then the dispersion and microscopic morphology of the different additives in the drilling fluids were observed through an optical microscope CX23 (Olympus Co., Ltd., Tokyo, Japan). Optical microscopy allows observation of the presence of weak gel mesh structures within the drilling fluid, and the measurement parameter is an image of the microscopic morphology of the drilling fluid with a size of 10 μm.

## Data Availability

Not applicable.
